# An NGS-assisted diagnostic workflow for culture-independent detection of bloodstream pathogens and prediction of antimicrobial resistances in sepsis

**DOI:** 10.3389/fcimb.2025.1656171

**Published:** 2025-09-01

**Authors:** David Pinzauti, Manuele Biazzo, Christine Podrini, Antonia Alevizou, Asimina Safarika, Georgia Damoraki, Panagiotis Koufargyris, Elisavet Tasouli, Ilias Skopelitis, Garyfallia Poulakou, Styliani Sympardi, Evangelos J. Giamarellos-Bourbolis

**Affiliations:** ^1^ The BioArte Limited, San Gwann, Malta; ^2^ 4th Department of Internal Medicine, National and Kapodistrian University of Athens, Medical School, Athens, Greece; ^3^ 1st Department of Internal Medicine, Thriaseio Geniko Nosokomeio Elefsinas, Magoula, Greece; ^4^ 3rd Department of Internal Medicine, Geniko Nosokomeio Nikaias Peiraia Agios Panteleimon, Nikaia, Greece; ^5^ 3rd Department of Internal Medicine, National and Kapodistrian University of Athens, Medical School, Athens, Greece; ^6^ Hellenic Institute for the Study of Sepsis, Athens, Greece

**Keywords:** molecular diagnostics, sepsis, full-length 16S rRNA, metagenomic sequencing, taxonomy, antimicrobial resistance, Oxford Nanopore technology

## Abstract

**Background:**

Timely and accurate identification of bloodstream pathogens is critical for targeted antimicrobial therapy in sepsis. Conventional blood cultures remain the Standard-of-Care (SoC) for pathogen identification but are limited by low sensitivity and prolonged turnaround times, hampering timely and targeted antimicrobial stewardship. Advances in next-generation sequencing (NGS) offer potential for culture-independent, rapid, and comprehensive detection of pathogens and prediction of antimicrobial resistance. This study evaluated the diagnostic performance of PISTE™ technology, an NGS-based diagnostic workflow combining full-length 16S rRNA gene sequencing and metagenomic analysis for the diagnosis of circulating bacteria in sepsis.

**Methods:**

In this prospective, multicenter, phase IIa proof-of-concept study, adult patients with suspected sepsis were enrolled from four hospitals in Athens, Greece. Blood samples were collected prior to antibiotic initiation and processed using SoC cultures and PISTE platform. PISTE integrates automated DNA purification (KingFisher, Thermo Fisher Scientific), full-length 16S rRNA gene sequencing, metagenomics analysis (SQK-PRB114.24, Oxford Nanopore Technologies), and real-time sequencing using Oxford Nanopore GridION Mk1b device. A dedicated analysis pipeline was developed for accurate pathogen detection and prediction of antimicrobial resistance profiles. The primary endpoint was the diagnostic concordance between PISTE and SoC cultures.

**Results:**

A total of 100 patients (median age 79 years, median Charlson’s Comorbidity Index 5) were enrolled. Of these, 71 patients met Sepsis-3 criteria. In this subgroup, PISTE showed an overall accuracy of 95.7%, with a sensitivity of 91.7%, specificity of 96.5%, positive predictive value of 84.6%, and negative predictive value of 98.2% compared to SoC. The median time to pathogen identification and Antimicrobial Susceptibility Testing (AST) with PISTE was 12.0 hours, significantly faster than in SoC cultures (30.4 hours, p < 0.0001). Resistance gene profiling showed strong agreement with SoC AST results, particularly for β-lactam and carbapenem resistance.

**Conclusions:**

PISTE technology exhibited high diagnostic accuracy and significantly reduced turnaround time compared to conventional cultures, supporting its potential as a short turnaround time and reliable diagnostic tool for bloodstream infections. Further optimization and validation in larger cohorts are warranted to enhance clinical implementation and improve antimicrobial stewardship in sepsis management.

## 1 Introduction

Sepsis is a life-threatening condition caused by a dysregulated immune response to infection, leading to organ dysfunction and high mortality rates. According to a recent global estimate (Rudd et al., 2020), sepsis accounted for approximately 48.9 million cases and 11 million deaths, representing nearly 20% of all global deaths. Early detection of microbial pathogens and start of antibiotics within the first 3 hours are the cornerstone of management (Kumar et al., 2006; Giamarellos-Bourboulis et al., 2017). However, the selection of antibiotics for early intervention is empirical and may be hampered by the emergence of antimicrobial resistance leading to ineffective treatment and increasing likelihood of adverse outcomes (Lamy et al., 2020). Currently available diagnostic methods frequently fail to provide actionable results within this critical window, limiting the ability to deliver targeted therapy. The only way to overcome this difficulty is guidance through early and rapid microbial identification and antibiotic susceptibility testing.

Conventional diagnostic methods, such as blood culture (BC) and Antimicrobial Susceptibility Testing (AST) remain the Standard-of-Care (SoC) in microbiology. However, these methods suffer from slow turnaround times delaying as much as 72 hours (Giamarellos-Bourboulis et al., 2017; Shi et al., 2024). SoC microbiology cultures workflows, consist of incubating whole blood into nutrient-rich flasks, followed by subculturing when a flask turns positive. Sepsis diagnosis based on microbial growth typically requires 16–48 hours for species identification and an additional 16–24 hours for AST (Shi et al., 2024). Moreover, prior antibiotic therapy or the presence of fastidious, slow-growing pathogens may lead to false negative results. It is evident that rapidly identify pathogens and perform AST directly from whole blood, without the need for a culture step, would represent a significant advancement in diagnostic microbiology.

Next-generation sequencing (NGS) technologies have emerged as a transformative tool for the rapid and comprehensive detection of microbial species. Unlike culture-based methods, NGS enables unbiased identification of bacterial, viral, and fungal pathogens directly from samples, offering a broader and more sensitive approach to sepsis diagnosis. Recent improvements in metagenomic sequences and newer sequencing strategies have significantly reduced the time to diagnosis supporting earlier, data-driven clinical decisions (Di Pilato et al., 2025). This is critical for a prompt and effective antimicrobial stewardship in sepsis patients, thereby enhancing the feasibility of NGS for clinical diagnostics (Lee et al., 2022; Wei et al., 2024; Liu et al., 2025).

In this study, we used an NGS-based workflow named PISTE™ (Pathogen Identification and quantification Sequencing Technology) to detect microbial pathogens from whole blood samples of patients with bloodstream infection. The aim was to investigate the potential of a novel NGS approach for the diagnosis of circulating pathogens in blood. Two sequencing strategies were adopted: i) full-length 16S rRNA amplicon sequencing approach allowing fast (~6h) and accurate microbial species characterization and ii) Metagenomic sequencing enabling prediction of antimicrobial resistance profiles. The primary objective of the study was to evaluate the diagnostic performance of NGS-based approach in comparison to “gold standard” SoC cultures in patients with sepsis.

## 2 Materials and methods

### 2.1 Study design

**Figure 1:**
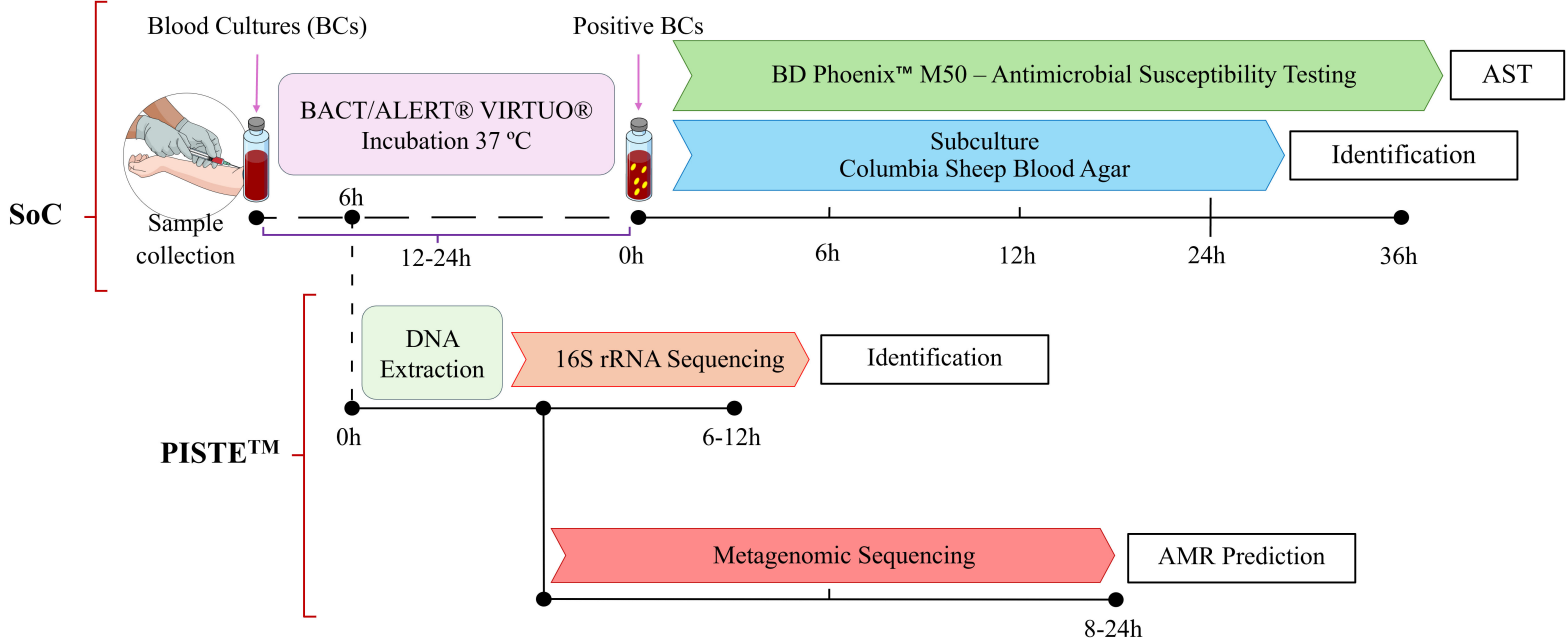
Study workflow. Schematic representation of the experimental workflow used in this study. Blood samples were processed using conventional SoC blood cultures (BCs) for pathogen identification and Antimicrobial Susceptibility Testing (AST). After 6 hours incubation, aliquotes from BCs were collected and analyzed using proprietary PISTE workflow. Following DNA extraction, a full-length 16S rRNA sequencing approach was applied to identify microbial species. In parallel, a Metagenomic sequencing strategy was used to detect Antimicrobial Resistance (AMR) genes and predict resistance profiles. The time ranges shown (e.g. 6–12 hours for 16S rRNA sequencing and 8–24 hours for Metagenomics) are approximate as actual turnaround times are influenced by sequencing runtime, microbial load, and available computational resources (data interpretation). Scientific icons were provided by Science Figure (https://sciencefigures.org).

This was a prospective, multicenter, proof-of-concept phase IIa clinical study in patients with severe infections conducted in collaboration between four study sites in Athens (Greece) and The BioArte Limited (San Gwann, Malta). A schematic representation of the study is represented in **Figure 1**. The study was approved by the Ethics Committees of the participating study sites (**Supplementary Table 1**). The study was conducted in accordance with the principles of the Declaration of Helsinki, Good Clinical Practice (GCP), and applicable regulatory requirements ClinicalTrials.gov ID NCT06141395 (sponsor Hellenic Institute for the Study of Sepsis).

### 2.2 Study population

Written informed consent was obtained from each patient or their legally designated representative prior to the initiation of any study procedures. Eligible participants included admitted adult patients of both sexes with a high suspicion of infection, defined by the presence of at least one sign of qSOFA (quick Sequential Organ Failure Assessment) score and for whom blood cultures were clinically indicated. Patients who had received any dose of antibiotics for the current infection episode, pregnant and breast-feeding women were excluded. During follow-up, data collected per patient included severity scores qSOFA and SOFA, Charlson’s comorbidity index, blood tests (total blood cell count, biochemistry and blood gases), infection type, and sepsis status (Sepsis-3 definition (Singer et al., 2016)). At the end of the study, SoC culture results were adjudicated by two blinded infectious disease experts using full clinical data to determine true pathogens versus putative contaminants.

### 2.3 Study interventions

For each enrolled patient, 20ml of whole blood were drawn for three consecutive days. The first sampling was performed before administration of the first dose of antibiotics. Blood samples were inoculated into one flask with growth medium for culture (Becton, Dickinson and Company, Sparks, USA) and immediately transported via courier within one hour to the central laboratory. Each flask was then placed into one autoincubator (BD) at 37°C. After six hours of incubation an aliquot of 5.5ml was removed under sterile conditions, stored at -80°C and shipped to The BioArte Ltd laboratories (San Gwann, Malta) for NGS analysis. Remaining blood culture (~14.5ml) was kept at 37°C and monitored for microbial growth to identify bacterial species and perform AST.

### 2.4 Standard-of-care workflows

Growth flasks (BACT/ALERT^®^ FA Plus; bioMérieux, Marcy-l’Étoile, France) were incubated at 37°C in a BACT/ALERT^®^ VIRTUO^®^ automated incubator (bioMérieux, Marcy-l’Étoile, France). Time to positivity, defined as the interval between the initiation of incubation and detection of bacterial growth by the automated system, was recorded. Upon detection of growth, the flask content was subcultured on COLUMBIA Sheep Blood Agar plates (Bioprepare Microbiology, Attica, Greece) and used for microbial identification and AST using the BD Phoenix™ M50 Automated Microbiology System (Becton, Dickinson and Company, Sparks, USA) with the following components: BD Phoenix™ ID Broth, BD Phoenix™ PMIC/ID-88, and BD Phoenix™ NMIC/ID-503. If any signal of bacterial growth was recorded after 7 days, the flask content was aspirated and cultured to exclude the presence of slow-growing pathogens.

### 2.5 PISTE workflow: DNA extraction, library preparation and sequencing

Total DNA was isolated from 0.5ml whole blood after 6 hours of incubation using the MagMax Microbiome Ultra II kit (Applied Biosystem, Waltham, Massachusetts, USA) and King Fisher device (Thermo Fisher Scientific). Samples were lysed through a bead-beating approach using the homogenizer MP FastPrep-24 5G (MP Biomedical) as follows: two cycles at 6.5 m/s for 45 sec each with a 5 minutes interval during which the samples were stored at 4°C. Two sequencing libraries based on Oxford Nanopore technology were prepared. The full-length 16S rRNA amplicon sequencing library was prepared as previously described (Pinzauti et al., 2024), introducing minor adjustments. Amplification was carried out in 25µL total volume pipetting 12.5µL Q5 Hot-Start High Fidelity 2x Master Mix (New England Biolabs), 4.5µL crude DNA extract, 400nM primers mix (Pinzauti et al., 2024), and 4µL nuclease-free water. The PCR reaction was carried out in a T100 thermal cycler (BioRad) using the following program: initial denaturation at 95°C for 3 minutes; followed by 32 cycles at 95°C for 20 s, 55°C for 30 s and 72°C for 2 minutes; and a final extension 72°C (5 minutes). PCR products were run on a 2% Agarose gel and cleaned with 0.6x Agencourt AMPure XP beads (Beckman Coulter). Barcoding PCR (sample multiplexing) was performed as follows: 12.5µL Q5 Hot-Start High Fidelity 2x Master Mix were mixed with 0.5µL phosphorylated barcoding primers mix (Pinzauti et al., 2024) and 100fmol 16S-amplicons from previous reaction (final reaction volume 25µL). The reaction was incubated at 95°C for 3 minutes (initial denaturation) followed by 15 cycles at 95°C for 15 s, 62°C for 15 s and 72°C for 2 minutes; and a final extension 72°C (5 minutes). Barcoded amplicons were purified with 0.6x AMPure XP beads, pooled together (equimolar pool), and adapter ligated following SQK-LSK114 (Oxford Nanopore Technologies) instructions. Briefly, 12.5µL Ligation Buffer (LNB), 5µL Quick T4 DNA Ligase (New England Biolabs), and 2.5µL Ligation Adapter (LA) were added to pooled barcodes (500ng DNA in 30µL). The reaction was incubated 10 minutes at room temperature. Clean-up was performed using 0.4x AMPure XP beads (AXP) washing twice with 250µL of Short Fragment Buffer (SFB) and resuspending in 15µL elution buffer (EB). Finally, 20fmol of DNA library were gently loaded in a dropwise manner onto a R10.4.1 flow cell (Oxford Nanopore Technologies) and sequenced using GridION Mk1b device (Oxford Nanopore Technologies). A minimum sequencing runtime of 2h was necessary to generate sufficient data.

The 16S-positive samples, identified by a sharp and clean band of ~1,500 bases in length during gel electrophoresis, were then subjected to Metagenomic sequencing. Metagenomics library was prepared following the Rapid PCR barcoding kit SQK-PRB114.24 (Oxford Nanopore Technologies). In short, 3µL crude DNA extract (expected 1 to 5ng DNA) were mixed with 1µL Fragmentation Mix (FRM) and incubated 2 minutes at 30°C followed by 2 minutes at 80°C (T100 thermal cycler, BioRad). Barcoding PCR reaction was prepared mixing 25µL LongAmp Taq 2x MasterMix (New England Biolabs), 20µL nuclease-free water, 4µL Tagmented DNA, and 1µL Rapid Barcode Primer mix (RLB, 10µM) in a clean 0.2ml thin-walled PCR tube. Reaction thermal profile consisted of an initial denaturation at 95°C for 3 minutes, followed by 25 cycles at 95°C for 15 s, 56°C for 15 s and 65°C for 6 minutes; and a final extension at 65°C for 6 minutes. Reaction was stopped by adding 4µL EDTA and incubating for 5 minutes (room temperature). Amplified samples were run on a 2% Agarose gel (expected sharp band of ~2,000 bases) and quantified using Qubit fluorometer. Barcoded samples were subsequently mixed generating an equimolar pool of 200–400 fmoles, purified with 0.6x AMPure XP beads (AXP), and resuspended in 15µL Elution Buffer (EB). Following, 10–50 fmol (20 to 100 ng DNA) of pooled library were transferred into a clean 1.5 ml Eppendorf DNA LoBind tube and mixed with 1µL diluted Rapid Adapter (RA), gently mixed and incubated for 5 minutes at room temperature. The library was gently loaded onto a R10.4.1 flow cell (Oxford Nanopore Technologies) mixing 37.5µL Sequencing Buffer (SB), 25.5µL Library Beads (LIB), and 12µL DNA library.

### 2.6 PISTE workflow: data analysis

Raw sequencing data were live basecalled and demultiplexed using Guppy v. 6.1.5 and super accurate mode (quality threshold > 12) through the GridION Mk1b platform. Sequencing yields were evaluated using NanoPlot v. 1.41.0 (De Coster and Rademakers, 2023) measuring overall read distributions and statistics. 16S amplicons were analysed as previously described (Pinzauti et al., 2024). Briefly, sequencing reads were trimmed and filtered using cutadapt v. 3.5 (Martin, 2011) and dada2 v. 1.28.0 (Callahan et al., 2016), while chimeric reads were pruned using minimap2 v. 2.16 (Li, 2018) and yacrd v. 1.0.0 (Marijon et al., 2020) tools. Taxonomy was inferred employing emu v. 3.4.5 (Curry et al., 2022) and the Genome Taxonomy Database (GTDB, release v. 207, accessed on March 2023 (Parks et al., 2022)). Metagenomics data were first screened for human host DNA contamination. FastQ reads were mapped against reference human database T2T-CHM13 v2.0 (Nurk et al., 2022) with minimap2 (-ax map-ont preset, -f 1000) (Li, 2018); any mapped reads were removed using samtools v1.21 (samtools view -b -f 4) (Danecek et al., 2021). The remaining microbial reads were then taxonomically classified using Kraken2 v. 2.1.0 (Wood et al., 2019) and the PlusPF-16 database, a catalog of complete genomes from NCBI RefSeq including archea, bacteria, virus, fungi, fungi, protozoa and human DNA capped at 16GB (https://benlangmead.github.io/aws-indexes/k2, accessed on 9 October 2023). Classified species were further refined using Bracken v. 2.9 (Lu et al., 2017). Potential Antimicrobial Resistance (AMR) genes were predicted using abricate v. 1.0.1 (https://github.com/tseemann/abricate): reads were *de novo* assembled using Flye v. 2.9.5 (–meta preset) (Kolmogorov et al., 2020) and resulting contigs were mapped against The Comprehensive Antibiotic Resistance Database (Jia et al., 2017). Detected AMR genes were further screened for clinical relevance: only genes conferring resistance to antimicrobials used in clinical and hospital settings, as defined by the World Health Organization Medically Important Antimicrobials List for Human Medicine (WHO MIA, https://cdn.who.int/media/docs/default-source/gcp/who-mia-list-2024-lv.pdf?sfvrsn=3320dd3d_2), were reported.

### 2.7 Statistical analysis and sample size

The study was not powered for the primary endpoint since this was a proof-of-concept phase IIa study. Since the rate of positivity of SoC cultures was expected to be 20% (Hadziyannis et al., 2004; Previsdomini et al., 2012), 100 patients were enrolled. Statistical analyses were performed with IBM SPSS Statistics for Windows (Version 29.0.2.0 Armonk, NY: IBM Corp) evaluating the classification matching rate between the 16S rRNA gene sequencing and the SoC cultures for patients classified with sepsis. Classification performance was evaluated by measuring the Sensitivity, the Specificity, the Positive Predictive Value (PPV), the Negative Predictive Value (NPV) and the Accuracy of the 16S rRNA sequencing approach. The aforementioned values were calculated as the ratios of True Positives/(True Positives + False Negatives), True Negatives/(True Negatives +False Positives), True Positives/(True Positives + False Positives), True Negatives/(True Negatives + False Negatives) and (True Positives + True Negatives)/(True Positives + True Negatives + False Positives + False Negatives), respectively. For the evaluation of diagnostic performance, results from SoC cultures were used as the reference standard. SoC culture results with disagreements between adjudicators were excluded from analysis. The above-mentioned values were expressed with percentages, rounded to the first digit after decimal point, accompanied by their 95% Confidence Intervals (CIs). For the secondary endpoints, the time comparison was assessed with the Mann-Whitney U test, with the significance level at 5%. Continuous variables following normal distribution were expressed by the mean and SD. Continuous variables not following the normal distribution were expressed by the median and interquartile range (Q1-Q3). Binomial variables were expressed as absolute and percentage frequencies with 95% CIs.

## 3 Results

### 3.1 Patients characteristics

Patients (n=262) with suspected sepsis were assessed for eligibility from November 21st, 2023 to March 15th, 2024. Of these, 162 patients were excluded as they received antibiotic treatment. The remaining 100 patients were enrolled in this study, including 40 males and 60 females, with a median (Q1-Q3) age of 79 (68–86) years. The medical history conditions (comorbidities) reported for the study population were diabetes mellitus type 1 or 2 (29 patients), heart failure (16 patients), chronic renal disease (13 patients), chronic obstructive pulmonary disease (19 patients), dementia (28 patients), coronary heart disease (20 patients), atrial fibrillation (25 patients), depression or psychosis (20 patients), residency in long-term care facility (14 patients), and intake of antimicrobials for any reason the last three months (19 patients). The median (Q1-Q3) Charlson’s comorbidity index score registered for all patients was 5. Regarding the type of infection, 8 patients were enrolled with primary bacteremia, 56 patients with community-acquired pneumonia (CAP), 2 patients with hospital-acquired pneumonia (HAP), 9 patients with acute biliary tract infections and 25 patients with acute pyelonephritis. The median (Q1-Q3) SOFA score at day 1 was 3 (Kumar et al., 2006; Giamarellos-Bourboulis et al., 2017; Lamy et al., 2020; Rudd et al., 2020; Shi et al., 2024). In total, 71 out of 100 enrolled patients met Sepsis-3 definition.

### 3.2 Comparison of diagnostic performance

**Figure 2:**
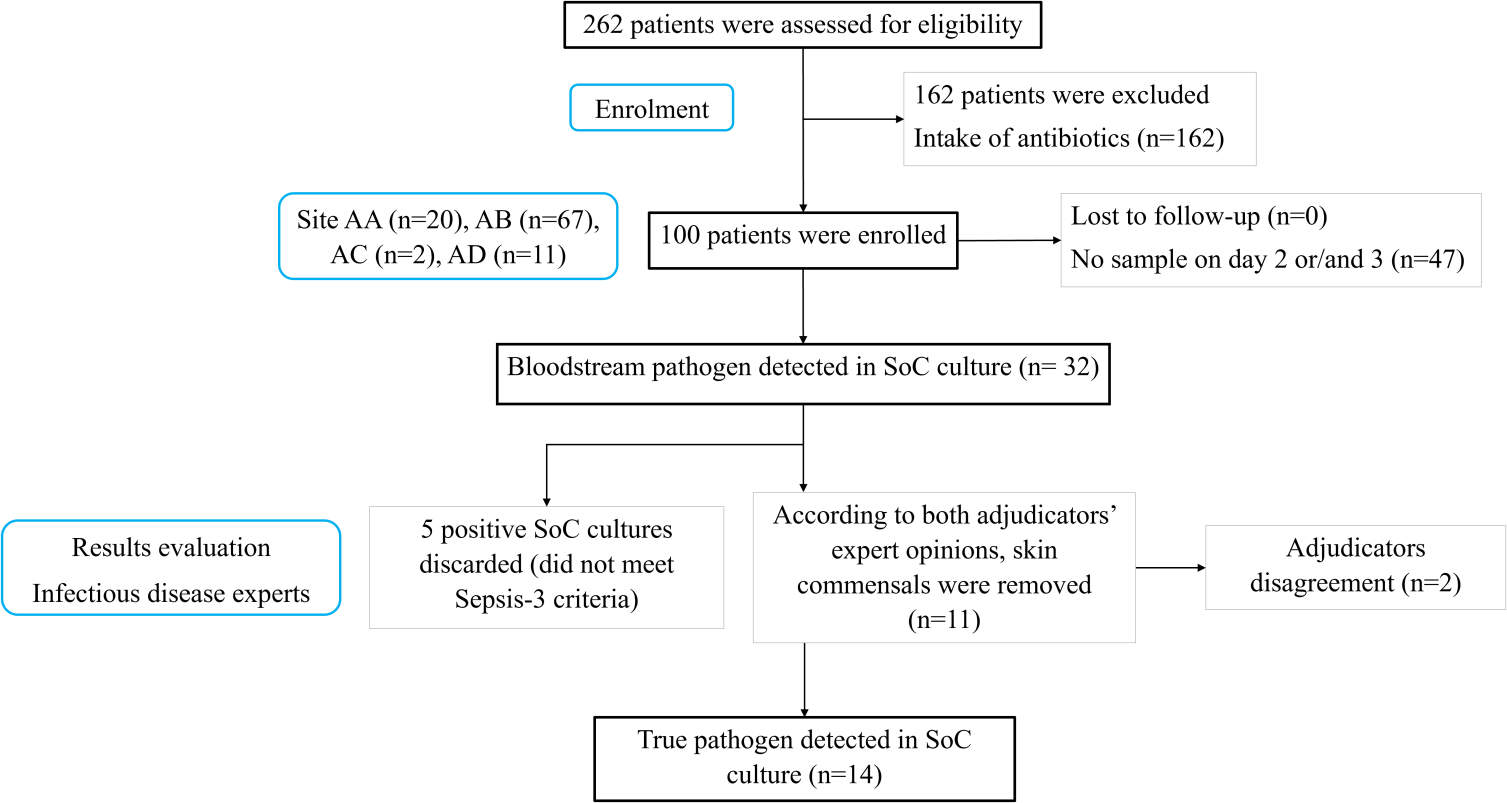
Patient recruitment overview. AA, Attikon University Hospital; AB, Thriaseio General Hospital; AC, Nikaia General Hospital; AD, Sotiria Thoracic Diseases General Hospital; SoC, Standard-of-Care; n, number of samples.

SoC blood cultures detected 32 positives over the three consecutive follow-up days. According to both adjudicators, 11 of these cases involved skin commensals rather than true pathogens and were excluded. Additionally, the adjudicators disagreed on 2 cases and therefore were discarded for the primary endpoint analysis. A further 5 samples were excluded as the patients did not meet the Sepsis-3 criteria for sepsis positivity. In total, true pathogens were detected in 14 patients by SoC cultures (**Figure 2**). The list of positive samples is provided in **Table 1**. According to microbiological data, a panel of 17 antimicrobials was tested evaluating sensitivity or resistance profiles. Because patient BAB002 failed species-level classification, it was not processed for AST. The list of tested antimicrobial compounds and their relative resistance profiles are listed in **Supplementary Table 2**.

**Table 1:** List of positive samples.

Patient	SoC blood culture	PISTE taxonomy
BAA002	*Escherichia coli*	*Escherichia coli*
BAA004	*Staphylococcus aureus*	*Staphylococcus hominis*
BAA018	*Escherichia coli*	*Escherichia coli*
BAA019	*Escherichia coli*	*Escherichia coli*
BAB002	*Enterococcus* spp.	–
BAB010	*Escherichia coli*	*Escherichia coli*
BAB014	*Proteus mirabilis*	*Proteus mirabilis*
BAB029	*Pseudomonas aeruginosa*	*Pseudomonas aeruginosa*
BAB037	*Klebsiella pneumoniae*	*Acinetobacter baumannii* *Klebsiella pneumoniae*
BAB041	*Proteus mirabilis*	*Proteus mirabilis*
BAB042	*Providencia stuartii*	*Providencia stuartii* *Staphylococcus hominis*
BAB046	*Acinetobacter baumannii*	*Acinetobacter baumannii*
BAB056	*Klebsiella pneumoniae*	*Klebsiella pneumoniae*
BAD003	*Escherichia coli*	*Escherichia coli*

The table lists all patients with microbiologically confirmed sepsis according to SoC cultures, alongside the microbial species identified by PISTE full-length 16S rRNA sequencing method. Discrepancies in microbiological species classification between the two methods are included (patients BAA004, BAB037, BAB042). “-” indicates no detection by PISTE technology.

**Figure 3:**
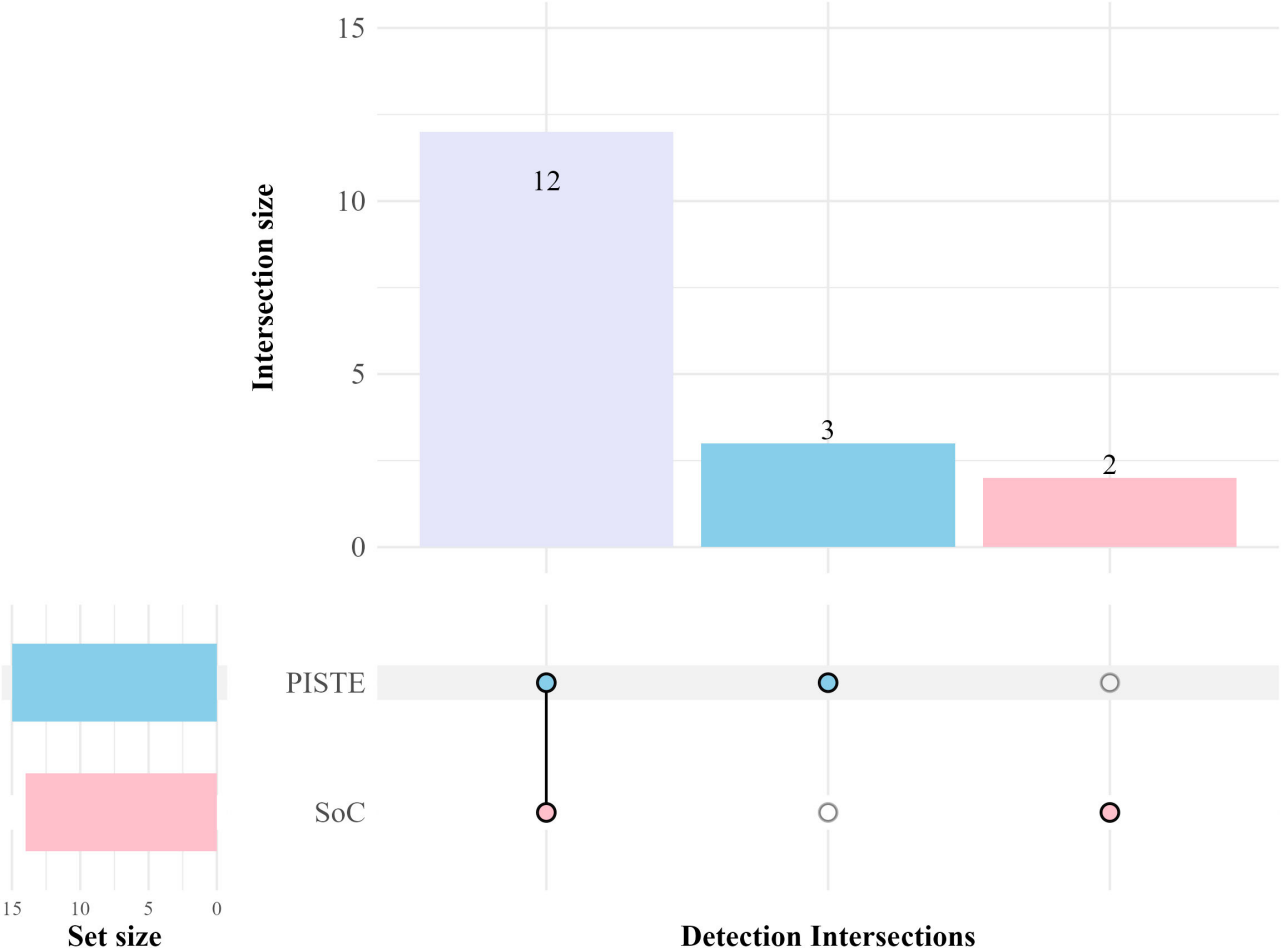
UpSet plot showing the shared and unique microbial detections between SoC and PISTE diagnostic methods. Each bar in the intersection matrix represents the number of microbial species uniquely identified by SoC (pink), by PISTE (light blue), or by both methods (light purple). Dots and connecting lines in the matrix indicate the specific diagnostic method(s) contributing to each intersection. Plot was generated with ComplexUpset v. 1.3.6 R package (Lex et al., 2014).

PISTE full-length 16S rRNA sequencing successfully detected 13 out of 14 SoC positives (**Table 1**; **Figure 3**). Patient BAB002 failed proper 16S gene amplification resulting in the absence of a visible, clear fluorescent band during gel electrophoresis. Nevertheless, the sample was processed through the entire sequencing library preparation as described but no sequencing data were generated, thus failing pathogen identification. Interestingly, PISTE workflow detected the presence of two microbial pathogens in patients BAB037 and BAB042. Specifically, in patient BAB037 the NGS-based analysis identified the pathogens *Klebsiella pneumoniae* (concordant with SoC cultures) and *Acinetobacter baumannii* (undetected by SoC cultures), while *Providencia stuartii* (concordant) and *Staphylococcus hominis* (undetected) were identified in patient BAB042. The overall concordance between the PISTE and SoC cultures demonstrated an overall 95.7% Accuracy (95% CI: 88-98.5), 91.7% Sensitivity (95% CI: 64.6-98.5), 96.5% Specificity (95% CI: 88.1-99), 84.6% PPV (95% CI: 57.8-95.7), and 98.2% NPV (95% CI: 90.6-99.7).

**Figure 4:**
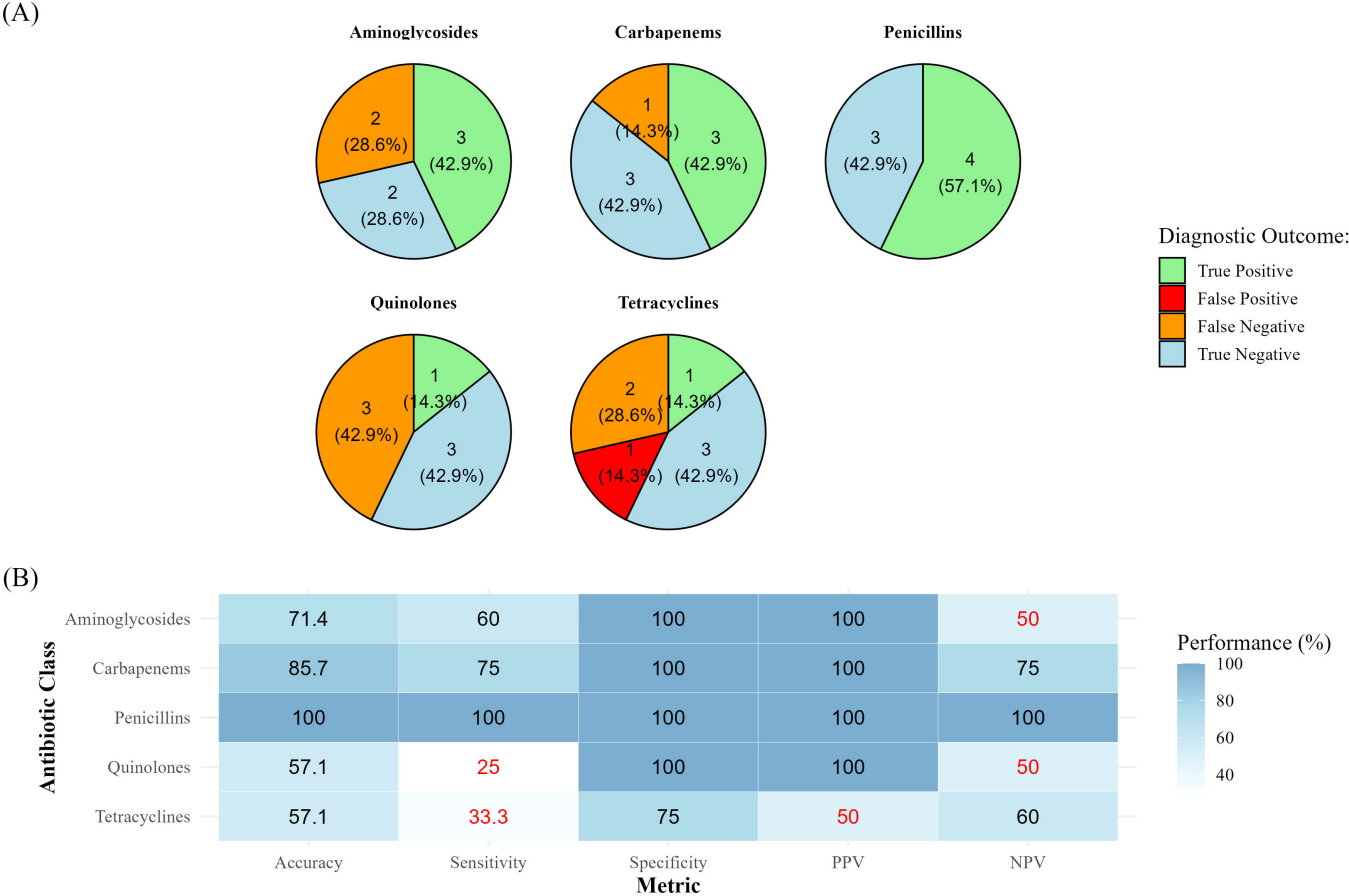
Diagnostic performance of PISTE compared to SoC for antimicrobial resistance detection. **(A)** Pie charts display the distribution of diagnostic outcomes for each antibiotic class tested. Each slice is annotated with the absolute count and corresponding percentage of measured True Positive (light green), False Positive (red), False Negative (orange), and True Negative (light blue) values. **(B)** Heatmap summarizing the diagnostic performance of PISTE expressed as Accuracy, Sensitivity, Specificity, Positive Predictive Value (PPV), and Negative Predictive Value (NPV). All metrics are expressed as percentages (%). Values below or equal 50% performance are visually labelled in red.

The 16S-positive samples were then processed through Metagenomics strategy prior antimicrobial resistance prediction. Of the 13 positive samples, only 7 (BAA002, BAB029, BAB037, BAB041, BAB042, BAB046, and BAD003) were successfully amplified and generated sufficient sequencing data. The remaining samples (6/13) likely failed due to insufficient microbial DNA, reflecting a low microbial biomass below technology detection threshold. Bracken classification showed 100% agreement with 16S-based taxonomy, further highlighting the presence of two microbial species in samples BAB037 and BAB042. Detected AMR genes (CARD database) were manually screened for the presence of relevant genes and compared to standard AST results for the tested antimicrobial compounds including Aminoglycosides, Cephalosporins, Carbapenems, Penicillins, Quinolones, and Tetracyclines (**Figure 4**). Resistance to Penicillins and Cephalosporins was marked by the detection of any of the following *bla*_TEM-1_, *bla*_CTX-M-15_, *bla*_CTX-M-27_, *bla*_CTX-M-139_, *bla*_OXA-1_, *bla*_OXA-10_, *bla*_OXA-66_, *bla*_SHV-182_, or *bla*_Z_ genes. Carbapenem resistance was predicted based on the identification of Metallo-β-lactamases *bla*_NMD-1_ and *bla*_VIM-1_, or other carbapenemases like *bla*_KPC_ and *bla*_OXA_, or multidrug efflux pump *mexA* and *mexB* genes. Detection of genes ACT-like *aac(6’)-lb10*, *aac(6’)-lb-cr*, and NUT-like *aadA2*, *aadA11* were used to predict Aminoglycoside resistance. Tetracycline resistance was inferred by the identification of *tetK* gene and efflux pump *mexA* and *mexB* genes, while Quinolone resistance was identified through detection of *qnrA*, *qnrB*, and *qnrS* genes. Respective cross-tabulations of the two assays for AST are presented in **Tables 2A-E** (**Figure 4A**). Antimicrobial resistance prediction showed 100% Accuracy for Cephalosporins and Penicillins (95% CI: 64.6-100), 85.7% for Carbapenems (95% CI: 48.7-97.4), and 71.4% for Aminoglycosides (95% CI: 35.9-91.8). Lower precision was obtained for Tetracyclines and Quinolones resistances (57.1% Accuracy, 95% CI: 25-84.2) (**Figure 4B**). The analysis of the turnaround times revealed a significant benefit in favor of the PISTE technology (*P* value < 0.0001). The NGS-based assay provided pathogen identification and AST at a fixed timepoint of 12.0 hours, while SoC cultures required a median of 30.4 hours (Q1-Q3: 28.9-33.7) to pathogen identification and AST reporting. However, reported time ranges were approximate as actual turnaround times are influenced by sequencing runtime, microbial load, and available computational resources (data interpretation).

**Table 2:** Cross-tabulation of antimicrobial resistance by PISTE and SoC cultures.

(A) Recorded resistance to Penicillins/Cephalosporins			(B) Recorded resistance to Carbapenems		
	SoC pos	SoC neg		SoC pos	SoC neg
PISTE pos	4	0	PISTE pos	3	0
PISTE neg	0	3	PISTE neg	1	3
(C) Recorded resistance to Aminoglycosides			(D) Recorded resistance to Tetracyclines		
	SoC pos	SoC neg		SoC pos	SoC neg
PISTE pos	3	0	PISTE pos	1	1
PISTE neg	2	2	PISTE neg	2	3
(E) Recorded resistance to Quinolones					
	SoC pos	SoC neg			
PISTE pos	1	0			
PISTE neg	3	3			

Each panel (A-E) presents diagnostic performance metrics for specific antibiotic classes, including Penicillins/Cephalosporins (A), Carbapenems (B), Aminoglycosides (C), Tetracyclines (D), and Quinolones (E). “SoC pos” and “PISTE pos” indicate samples classified as resistant; “SoC neg” and “PISTE neg” indicate samples classified as sensitive.

## 4 Discussion

The application of NGS in clinical diagnosis is of great interest, particularly in time-sensitive conditions such as sepsis. Multiple culture-independent diagnostic approaches have emerged in recent years to accelerate sepsis diagnosis compared to blood cultures. Molecular tools such as multiple PCR panels, T2 Magnetic Resonance (T2MR), and broad range 16S/18S PCR assays offer faster results (2 to 6 hours) by directly detecting pathogen DNA in blood without requiring culture (Banerjee et al., 2015; Samuel, 2023). However, many direct PCR-based assays still require high bacterial loads to achieve reliable detection due to host DNA contamination and are inherently limited by predefined pathogen and AMR panels. On the contrary, targeted broad-range PCR and sequencing allow species-level identification of virtually any bacterial or fungal organism, but they suffer from relatively poor sensitivity and are prone to contamination. In contrast, NGS represents a valuable all-in-one tool for pathogen identification and antimicrobial resistance prediction (Rhoads, 2018; Lee et al., 2022; Zhang et al., 2024; Di Pilato et al., 2025; Liu et al., 2025). While its implementation in clinical diagnosis is hampered by several barriers including high sequencing costs, slow turnaround time, cost, the need of host DNA depletion, and the lack of standardized bioinformatic pipelines (Peri et al., 2022), advances in sequencing technologies, particularly with real-time Oxford Nanopore, have made clinical NGS increasingly viable (Charalampous et al., 2019; Guo et al., 2023; Di Pilato et al., 2025). Guo et al. demonstrated that a dual-process pipeline combining 16S rRNA gene sequencing and shotgun metagenomics could rapidly (actionable results in under 8 hours) and accurately diagnose lower respiratory tract infections (Guo et al., 2023), while similarly Charalampous et al. showed that Oxford Nanopore metagenomics enabled pathogen detection directly from respiratory specimens within 6 to 8 hours, without the need for culture (33). Recently, Di Pilato and colleagues (Di Pilato et al., 2025) introduced an innovative whole-genome sequencing workflow named LC-WGS (Whole-Genome Sequencing of Liquid Colony). This workflow, which relies on the Qvella FAST system (Qvella) alongside the SQK-RBK004 Rapid Barcoding chemistry (Oxford Nanopore Technologies), achieved fast (2.6-4.2h), accurate pathogen detection - 98% of monomicrobial and 88% of polymicrobial infections - as well as antimicrobial resistance profiles prediction (94%) from positive blood cultures. The study represents strong and significant methodological advancements for the analysis of positive blood cultures in time-critical scenarios. However, the procedure entirely revolves around the Qvella FAST system (Qvella), a fully automated platform capable of isolating and concentrating pathogens while efficiently depleting any traces of cell-free DNA and host DNA. This dependence limits its applicability to laboratories or hospitals equipped with the Qvella system. Furthermore, the time it takes for blood cultures to become positive (12 to 24h) significantly impacts the diagnosis, delaying both pathogen identification and antimicrobial treatment. Building on these recent advancements, our study aimed to address some of these limitations by evaluating a novel, culture-independent sequencing-based approach for direct pathogen detection and AMR profiling from whole blood.

In this view, we introduce PISTE (Pathogen Identification and quantification Sequencing Technology), an Oxford Nanopore-based NGS workflow designed for accelerated microbial pathogens detection and prediction of antimicrobial resistance profiles from whole blood samples of patients with bloodstream infections. Compared to existing culture-independent diagnostic tools, which are limited by narrow detection panels or require prior culture positivity, PISTE offers untargeted pathogen identification and AMR profiling from blood samples. The workflow combines automated DNA extraction with KingFisher device from blood cultures after 6h incubation and Oxford Nanopore sequencing technology enabling full-length 16S rRNA sequencing (pathogen detection) and Metagenomic sequencing (antimicrobial resistance prediction). In contrast to SoC, which depends on blood culture positivity and typically takes 24–48 hours, the PISTE workflow reduces total diagnostic time to approximately 6.5 hours, enabling earlier clinical intervention. In our study cohort, 100 patients were enrolled and 71/100 (71%) were declared sepsis-positive (Giamarellos-Bourboulis et al., 2017). When it comes to microbial detection, by PISTE full-length 16S rRNA sequencing 95.7% Accuracy, 91.7% Sensitivity, and 96.5% Specificity were recorded compared to SoC cultures. The overall turn-around time was approximately 6.5 h compared to the 30.4 hours for SoC. Antimicrobial resistance was inferred through a secondary sequencing step involving Metagenomic sequencing strategy. Of the sepsis-positive samples, 7 (63.6%) were successfully sequenced and screened for resistance markers. PISTE workflow successfully enabled characterization of AMR genes associated with resistance to Cephalosporins, Penicillins, Carbapenems, and Aminoglycosides, although lower classification rates were achieved for Tetracyclines and Quinolones resistances. This study highlights the potential of PISTE workflow as a short turnaround time and reliable tool for identifying bloodstream bacteria and predicting their antimicrobial resistance profiles in a significantly shorter interim compared to SoC cultures. By enabling earlier targeted antibiotic administration, the workflow has the potential to meaningfully change the clinical management and care pathway of patients with severe infections. However, several limitations should be considered. The first limitation comes from the lack of a host DNA depletion during DNA isolation. Since human DNA dominates blood samples, effective DNA depletion represents a key point for future improvements enhancing detection sensitivity, reducing costs and turnaround time, limiting computational burden, and improving accuracy in pathogen and resistance genes detection. Another limitation arises from the adopted sequencing strategy. Moreover, further multicenter validation is required to assess reproducibility and generalizability across diverse patient populations and laboratory settings. In its current state PISTE workflow includes two library preparation phases: an initial screening through a full-length 16S rRNA amplicon sequencing approach for rapid and accurate bacterial detection, followed by a Whole-Genome Metagenomic sequencing to enable AMR genes prediction. Both strategies depend on PCR amplification and users may experience slower turn-around times (incubation times and multiple clean up stages) and issues derived from suboptimal PCR conditions (e.g. primer choice, thermal profiles, polymerases).

In the present study, we validated an analytical workflow based on DNA extraction from blood cultures after 6-hour incubation, followed by pathogens identification and resistance profiling through Oxford Nanopore sequencing. The PISTE workflow enabled fast and accurate results comparable to SoC methods, but in a significantly shorter timeframe. Additionally, the adopted long-read sequencing platform offers the potential to fully unravel genomic features of target pathogens (e.g. virulence factor, AMR genes, in silico serotyping and virulotyping, and phylogenomic) and provide a more detailed view of the genetic resistance markers detected (Di Pilato et al., 2025). Further studies are required to validate the clinical utility of the workflow. Optimization of DNA extraction methods, coupled with effective host DNA depletion, are strongly recommended to improve sensitivity and increase overall performance. Although phenotype-based Antimicrobial Susceptibility Testing remains the gold standard concerning antimicrobial resistance profiling, an integrated approach combining both NGS-based AMR detection and AST may enhance antimicrobial stewardship by ensuring faster and more precise treatment decisions. Taken together, our findings support the clinical potential of PISTE to expedite diagnosis and guide targeted antimicrobial therapy in sepsis. While further optimization and validation are necessary, this technology may bridge current diagnostic gaps, offering a faster, hypothesis-free alternative to conventional methods.

## 5 Conclusion

Timely and accurate identification of bloodstream pathogens is critical for targeted antimicrobial therapy in sepsis. Conventional SoC blood cultures remain the gold standard for microbial diagnosis but suffer from low sensitivity and prolonged turnaround times. In this proof-of-concept study we evaluated the diagnostic potential of PISTE technology, a culture-independent next-generation sequencing workflow that integrates full-length 16S rRNA sequencing with metagenomics. PISTE demonstrated high diagnostic accuracy and significantly faster results compared to SoC cultures, with strong agreement in resistance profiling. These findings support the potential of NGS technology as a powerful diagnostic tool for broad-spectrum surveillance and hypothesis-free identification of pathogens and resistance genes, enhancing early diagnosis and improving clinical decision-making in sepsis management (next-generation diagnosis). However, the current workflow still faces limitations. Further validation in larger, multicenter cohorts is necessary to confirm clinical utility, especially in diverse patient populations. In addition, integration of host DNA depletion methods and streamlined library preparation could further enhance sensitivity and scalability. These improvements will be critical for routine clinical adoption. By reducing time to diagnosis and appropriate antimicrobial therapy, the technology has the potential to improve patient outcomes and strengthened antimicrobial stewardship efforts.

## Data Availability

The datasets generated for this study can be found in the ENA (https://www.ebi.ac.uk/ena/browser/view/PRJEB89897) repository, accession number PRJEB89897.
