# Supplementary material for: An NGS-assisted diagnostic workflow for culture-independent detection of bloodstream pathogens and prediction of antimicrobial resistances in sepsis
Source: Front Cell Infect Microbiol. 2025 Sep 1;15:1656171. doi: 10.3389/fcimb.2025.1656171 (PMC12434068; doi:10.3389/fcimb.2025.1656171)
Supplement: Supplementary file 1 [file Table1.docx]

Supplementary Material

# Supplementary Table 1. List of approvals of the Ethics Committees.

| **Study site** | **Site Code** | **Approval of Ethics Committee** | **Date of Approval** |
| --- | --- | --- | --- |
| 4th Department of Internal Medicine, ATTIKON University Hospital | Site AA | Approval of the Ethics Committee of  the 12th Regular Meeting, 2023 | 12/09/2023 |
| 1st Department of Internal Medicine, Thriaseio General Hospital | Site AB | Approval of the Ethics Committee of  the 15th Meeting, 2023 | 13/09/2024 |
| 3rd Department of Internal Medicine, Nikaia General Hospital | Site AC | Approval No.4 of Ethics  Committee, September 6, 2023 | 06/09/2023 |
| 3rd Department of Internal Site Medicine, SOTIRIA Thoracic Diseases General Hospital, Athens | Site AD | Approval of the Ethics Committee  23204/15-09-2023 | 15/09/2023 |

# Supplementary Table 2. Antimicrobial Susceptibility Testing.

| **Patient** | **Species** | Amikacin | Amoxicillin/Clavulanate | Ampicillin | Cefepime | Ceftazidime | Ceftazidime/Avibactam | Ceftriaxone | Cefuroxime |
| --- | --- | --- | --- | --- | --- | --- | --- | --- | --- |
| **BAA002** | *E. coli* | Sensitive | Resistant | Resistant | Resistant | Resistant | Sensitive | Resistant | Resistant |
| BAA004 | *S. aureus* | Sensitive | Sensitive | Sensitive | Sensitive | Sensitive | Sensitive | Sensitive | Intermediate |
| BAA018 | *E. coli* | Sensitive | Resistant | Resistant | Resistant | Resistant | Sensitive | Resistant | Resistant |
| BAA019 | *E. coli* | Resistant | Resistant | Resistant | Resistant | Resistant | Sensitive | Resistant | Resistant |
| BAB002 | *Enterococcus* spp. |  |  |  |  |  |  |  |  |
| BAB010 | *E. coli* | Sensitive | Resistant | Resistant | Resistant | Resistant | Sensitive | Resistant | Resistant |
| BAB014 | *P. mirabilis* | Resistant | Sensitive | Sensitive | Sensitive | Sensitive | Sensitive | Sensitive | Intermediate |
| BAB029 | *P. aeruginosa* | Resistant | N/A | N/A | Resistant | Resistant | Resistant | N/A | N/A |
| BAB037 | *K. pneumoniae* | Resistant | Resistant | Resistant | Resistant | Resistant | Resistant | Resistant | Resistant |
| BAB041 | *P. mirabilis* | Sensitive | Sensitive | Sensitive | Sensitive | Sensitive | Sensitive | Sensitive | Intermediate |
| BAB042 | *P. stuartii* | Resistant | Resistant | Resistant | Resistant | Resistant | Resistant | Resistant | N/A |
| BAB046 | *A. baumannii* | Resistant | N/A | N/A | N/A | N/A | N/A | N/A | N/A |
| BAB056 | *K. pneumoniae* | Resistant | Resistant | Resistant | Resistant | Resistant | Sensitive | Resistant | Resistant |
| BAD003 | *E. coli* | Sensitive | Resistant | Resistant | Sensitive | Sensitive | Sensitive | Sensitive | Intermediate |

| **Patient** | Ciprofloxacin | Colistin | Ertapenem | Gentamicin | Imipenem/Cilastatin | Levofloxacin | Meropenem | Piperacillin/Tazpbactam | Tigecycline |
| --- | --- | --- | --- | --- | --- | --- | --- | --- | --- |
| **BAA002** | Intermediate | Sensitive | Sensitive | Resistant | Sensitive | Sensitive | Sensitive | Sensitive | Resistant |
| BAA004 | Sensitive | Sensitive | Sensitive | Sensitive | Sensitive | Sensitive | Sensitive | Sensitive | Resistant |
| BAA018 | Resistant | Sensitive | Sensitive | Resistant | Sensitive | Resistant | Sensitive | Resistant | Resistant |
| BAA019 | Resistant | Sensitive | Sensitive | Resistant | Sensitive | Resistant | Sensitive | Resistant | N/A |
| BAB002 |  |  |  |  |  |  |  |  |  |
| BAB010 | Intermediate | Sensitive | Sensitive | Sensitive | Sensitive | N/A | Sensitive | Sensitive | Resistant |
| BAB014 | Sensitive | Resistant | Resistant | Resistant | N/A | Sensitive | Sensitive | Sensitive | Resistant |
| BAB029 | Resistant | Sensitive | N/A | N/A | Resistant | N/A | Resistant | Resistant | N/A |
| BAB037 | Resistant | Resistant | Resistant | Resistant | Resistant | N/A | Resistant | Resistant | N/A |
| BAB041 | Sensitive | Resistant | Sensitive | Sensitive | N/A | Sensitive | Sensitive | Sensitive | Resistant |
| BAB042 | Resistant | Resistant | Resistant | Resistant | Resistant | Resistant | N/A | Resistant | Resistant |
| BAB046 | Resistant | Sensitive | N/A | Resistant | Resistant | Resistant | Resistant | N/A | N/A |
| BAB056 | Sensitive | Sensitive | Sensitive | Sensitive | Sensitive | N/A | Sensitive | Sensitive | N/A |
| BAD003 | Sensitive | Sensitive | Sensitive | Sensitive | Sensitive | Sensitive | Sensitive | Sensitive | Sensitive |

N/A, Antibiotic not tested for the specific pathogen.
